# The effect of local hydrodynamics on the spatial extent and morphology of cold-water coral habitats at Tisler Reef, Norway

**DOI:** 10.1007/s00338-017-1653-y

**Published:** 2017-12-19

**Authors:** L. H. De Clippele, V. A. I. Huvenne, C. Orejas, T. Lundälv, A. Fox, S. J. Hennige, J. M. Roberts

**Affiliations:** 1 School of Energy, Geoscience, Infrastructure and Society, Heriot-Watt University, Edinburgh, EH14 4AS UK; 2 Marine Geoscience, National Oceanography Centre, University of Southampton Waterfront Campus, European Way, Southampton, SO14 3ZH UK; 3 Instituto Español de Oceanografía, Centro Oceanográfico de Baleares, 07015 Palma, Mallorca Spain; 4 The Swedish Institute for the Marine Environment, University of Gothenburg, Gothenburg, Sweden; 5 School of Geosciences, King’s Buildings, West Mains Road, Edinburgh, EH9 3FE UK

**Keywords:** Tisler Reef, Hydrodynamics, Cold-water coral habitat, Morphology

## Abstract

This study demonstrates how cold-water coral morphology and habitat distribution are shaped by local hydrodynamics, using high-definition video from Tisler Reef, an inshore reef in Norway. A total of 334 video frames collected on the north-west (NW) and south-east (SE) side of the reef were investigated for *Lophelia pertusa* coral cover and morphology and for the cover of the associated sponges *Mycale lingua* and *Geodia* sp. Our results showed that the SE side was a better habitat for *L. pertusa* (including live and dead colonies). Low cover of *Geodia* sp. was found on both sides of Tisler Reef. In contrast, *Mycale lingua* had higher percentage cover, especially on the NW side of the reef. Bush-shaped colonies of *L. pertusa* with elongated branches were the most abundant coral morphology on Tisler Reef. The highest abundance and density of this morphology were found on the SE side of the reef, while a higher proportion of cauliflower-shaped corals with short branches were found on the NW side. The proportion of very small *L. pertusa* colonies was also significantly higher on the SE side of the reef. The patterns in coral spatial distribution and morphology were related to local hydrodynamics—there were more frequent periods of downwelling currents on the SE side—and to the availability of suitable settling substrates. These factors make the SE region of Tisler Reef more suitable for coral growth. Understanding the impact of local hydrodynamics on the spatial extent and morphology of coral, and their relation to associated organisms such as sponges, is key to understanding the past and future development of the reef.

## Introduction

Cold-water coral (CWC) ecosystems are protected in national and international waters. They are considered vulnerable marine ecosystems (United Nations 2006) and meet the criteria of ecologically or biologically significant areas (EBSAs) (CBD Secretariat 2012). However, there is a need to improve our understanding of what drives differences in the fine-scale spatial distribution of CWCs to protect these ecosystems effectively in the future. CWC reef distribution depends on the availability of hard substrate for settlement as well as on food supply in a highly dynamic environment (Wilson 1979; Frederiksen et al. 1992; White et al. 2005; Mienis et al. 2007; Davies et al. 2009; Roberts et al. 2009). Other environmental factors such as temperature, salinity, density and oxygen availability also play important roles in controlling their distribution (Roberts et al. 2009; Purser et al. 2010; Flögel et al. 2014).

Over a period of 2 yr, Wagner et al. (2011) studied hydrodynamic processes and how they affect food delivery to the reef at Tisler CWC Reef (Hvaler area, Norway). They found that Tisler Reef has a dynamic environment with average high current speeds of 10–50 cm s^−1^ and a peak current speed of 74 cm s^−1^. The flow direction on Tisler Reef alternates between north-westward and south-eastward. The hydrodynamics on Tisler Reef show similarities to those described for the Mingulay Reef Complex, offshore of Scotland, where downwelling occurs downstream of the reef (Duineveld et al. 2012), delivering chlorophyll-rich, warmer water (5.6–13.9 °C) from the surface to the bottom which can stimulate the growth of CWCs (Davies et al. 2009; Findlay et al. 2013). Compared to Mingulay Reef, the tidal flows in the Hvaler area are weak (5–10 cm s^−1^) (Lavaleye et al. 2009). The residual flow is wind and buoyancy driven, and its direction can stay constant for several days to several weeks before reversing (Lavaleye et al. 2009; Wagner et al. 2011). Since current flow direction alternates and downwelling occurs over downstream sill crests, this vertical flux occurs at both ends of Tisler Reef where it supplies food to the seabed, supporting benthic secondary productivity including coral growth. Wagner et al. (2011) demonstrated this by analysing near-bed current direction and velocity over a period of 2 yr and by measuring the temperature, chlorophyll *a* concentration, and salinity across the water column using acoustic Doppler current profiler (ADCP) and CTD casts on both the SE and NW sides of Tisler Reef (see Fig. 2 in Wagner et al. 2011).

Corals have different growth forms as they have a high level of phenotypic variation (Foster 1979; Bell and Barnes 2001; Todd 2008; Gori et al. 2013; Vad et al. 2017). Local hydrodynamics, which affect availability of food to the corals and their ability to feed over short (Purser et al. 2010; Orejas et al. 2016) and longer timescales, are an important determinant of coral morphology (Wainwright and Dillon 1969; Mortensen and Buhl-Mortensen 2005; Todd 2008). Branching corals, in particular, seem to respond to changes in hydrodynamics, with their shape becoming more compact in high current speeds, and more asymmetrical when currents are unidirectional. More symmetrical, open frameworks with thin branches form when the current speeds are lower (Kaandorp 1999; Chindapol et al. 2013). The morphology of a coral colony will also affect the availability of food to the polyps by altering small-scale turbulence and slowing current speeds in the immediate proximity of living polyps. Coral colonies that are less compact and have thinner branches are more likely to capture food particles as water will flow through them more easily than in compact colonies. When the current speeds are too high, a more compact morphology with thick branches creates more stability for the colony (Chamberlain and Graus 1975). Aside from local hydrodynamics, genetic differences and other variables such as the availability of food and sedimentation rates can cause variation in the growth forms of corals (Barnes 1973; Foster 1979; Willis and Ayre 1985; Smith et al. 2007).

The habitat that is created by the CWC framework is a place for organisms such as crabs, fish and sharks to feed, shelter and reproduce (Henry and Roberts 2007, 2017; Baillon et al. 2012; Buhl-Mortensen et al. 2017). The live and dead coral structures provide a substrate for benthic filter feeders such as sponges and crinoids as well as other cnidarians (Orejas and Jiménez 2017). The coral creates an elevated feeding platform, exposing the filter feeders to higher current speeds, increasing their chances of capturing food (Roberts 2005; Mortensen and Fosså 2006; Henry and Roberts 2007; Buhl-Mortensen et al. 2017). Sponges can occur in especially large numbers forming a substantial component of CWC reef biomass (Hogg et al. 2010). They function as nutrient recyclers, substrate stabilizers, bioeroders, as a food source and as a habitat for other organisms (Wulff 2001).

Wagner et al. (2011) found that particulate organic matter (POM) composition in the benthic boundary layer across Tisler Reef differed between the two sides of the reef. Downstream POM was fresher in composition than upstream samples. With the observation that downwelling occurs on both sides of Tisler Reef, coral and sponge growth are also expected to be supported on both sides of the reef. To test this null hypothesis, we examined the percentage cover of important ecosystem engineering species (the coral *L. pertusa* and two dominant sponges *M. lingua* and *Geodia* sp.) in relation to local hydrodynamics. We also assessed the densities of different coral growth morphologies on each side of the reef.

## Materials and methods

### Study area

**Fig. 1:**
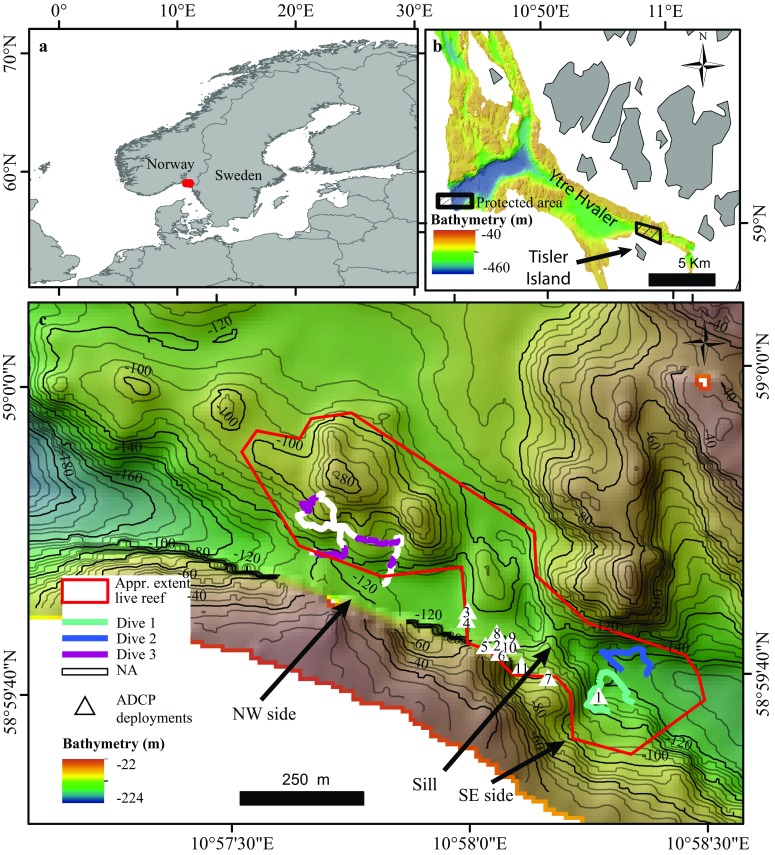
**a** Location of Tisler Reef in Norway. **b** Location of protected area surrounding Tisler Reef (black box) in Kosterfjord. **c** Locations of remotely operated vehicle dives. The white part of dive 3 represents data which was not available (NA) for analysis. The white triangles with numbers show locations of the acoustic Doppler current profiler (ADCP) deployments

CWCs at Tisler Reef were first discovered in 2002 (Lundälv and Jonsson 2003). The reef lies in the north-eastern part of the Skagerrak in the Hvaler area in Norway (Lavaleye et al. 2009) (Fig. 1). The reef is located north of Tisler Island, in a 48-km-long ocean channel through which Atlantic water flows in the Ytre Hvaler (Fig. 1b) (Guihen et al. 2012). The water temperature commonly ranges around 8 °C (Wagner et al. 2011), and the reef is thought to be between 8600 and 8700 yr old (Wisshak et al. 2005). The live part of the reef is approximately 70–160 m deep, 1.2 km long, 200 m wide and is oriented in a NW–SE direction (Fig. 1c) (Lavaleye et al. 2009). It extends both on and off the mound structures visible in the reef area (Fig. 1c). Remotely operated vehicle (ROV) examinations by T. Lundälv (HD video) indicated that the outcrops inside and outside the live reef are made up of both live and dead coral. Reefs that have developed after the last glacial maximum are typically smaller, depend strongly on the availability of settling substrates (strong substrate control) and owe their overall morphology to the features they colonized (e.g. Sula Ridge, Mingulay Reef Complex), and are therefore classified by Wheeler et al. (2007) as having an “inherited” morphology. To know whether the mound structures inherited the morphology of the bedrock, seismic data are necessary which is currently unavailable. In December 2003, Tisler Reef was protected against bottom-impacting fishing techniques by Norwegian fishery regulations (Fosså et al. 2010) (Fig. 1b).

### Hydrodynamic data

An RDI Workhorse 300-kHz ADCP was deployed eleven times to measure current speed and direction every 30 min, from 2006 to 2010, in different locations on the reef (Table 1; Fig. 1c). The ADCP deployments were all near the reef sill and not at the NW and SE ends, except for the first deployment ADCP1, which was close to dive 1 (Fig. 1c). The longest continuous logging, 8 months and 9 d, occurred during deployment ADCP10. ADPC11 only recorded for 3 d and is therefore not included in the analyses. The data were part of a larger environmental monitoring project at Tisler Reef (Lavaleye et al. 2009).

**Table 1:** The location, deployment time and depth for of acoustic Doppler current profiler (ADCP) deployments that were used to record measurements of current speeds and direction

Longitude	Latitude	Data name	Deployed	Recovered	Depth (m)	Days
10.97126	58.99413	LF ADCP 1	27/03/2006	27/04/2006	138	31
10.96785	58.99511	LF ADCP 2	04/05/2006	02/10/2006	111	181
10.96676	58.99573	LF ADCP 3	05/10/2006	29/04/2007	120	206
10.96670	58.99558	LF ADCP 4	30/04/2007	04/12/2007	121	218
10.96735	58.99510	LF ADCP 5	04/12/2007	15/04/2008	112	133
10.96785	58.99496	LF ADCP 6	15/04/2008	04/08/2008	117	111
10.96951	58.99448	LF ADCP 7	04/08/2008	23/02/2009	109	203
10.96776	58.99531	LF ADCP 8	25/02/2009	05/08/2009	119	161
10.96826	58.99518	LF ADCP 9	06/08/2009	11/11/2009	121	97
10.96813	58.99508	LF ADCP 10	12/11/2009	23/07/2010	113	253
10.96860	58.99471	LF ADCP 11	02/09/2010	05/09/2010	110	3

Water flow on Tisler Reef is channelled over a sill through the sound, which has a NW–SE orientation. The currents can flow in either direction, to the north-west or to the south-east. The amount of time that the current flowed in each direction was calculated using the data provided by ten of the ADCP deployments. The average flow speed was calculated per ADCP deployment; an annual average was unreliable because the currents vary with the position of the ADCP instrument (Fig. 1c). The ADCP data were binned at 2-m intervals every 20 min. Since instruments positioned at the seabed experience higher turbulence caused by friction with the sea floor and the reef itself, all ADCP data used for the calculations were recorded at 86 m depth, at least 20 m above the seafloor.

### Multibeam data

Bathymetry data were collected with a shipboard multibeam echosounder (SeaBeam 1050) during ALKOR cruise 232 in 2003. Data were processed using Fledermaus software resulting in a grid with pixel size of 8.22 × 8.22 m.

### Video data collection

The *R*/*V Lophelia* from Tjärnö Marine Biological Laboratory (TMBL, University of Gothenburg) was used to deploy the ROV Sperre SubFighter 7500 DC to record high-definition video transects at Tisler Reef on 22 and 23 May 2014. Two video transects were collected on the SE end (dive 1 and 2) with a length of 239 m and 203 m, respectively. On the NW end, a single transect was collected (dive 3) (Table 2) from which 57% of the data were not useful due to limited visibility caused by increased sediment resuspension and high variability in the vehicle’s altitude above the seafloor (Fig. 1). These transects were chosen as they were within the live reef area and closest to the NW and SE stations where Wagner et al. (2011) conducted their first two CTD, chlorophyll and POM measurements.

**Table 2:** High-definition video transects recorded on the SE and NW sides of Tisler Reef in 2014

Dive	Side	Start Lat	Start Lon	End Lat	End Lon	Depth range (m)	Average depth (m)	Length (m)	Time (min)
1	SE	58.99397	10.97252	58.99458	10.97224	124–142	135	239	41
2	SE	58.99455	10.97200	58.99487	10.97169	129–147	139	203	28
3	NW	58.99683	10.96219	58.99625	10.96381	77–130	110	622	50

*Side* side of the reef, *Lat* latitude, *Lon* longitude

A Sony FCBH11 HD camera with two Sperre 200-W HMI lights was used to collect the video footage from an altitude of ~ 1 m. Video signals were transmitted over an optical fibre and recorded on compact flash cards using a nanoFlash recorder (Convergent Design). The ROV moved at an average speed of 0.7 knots. Two laser beams, spaced by 5 cm, were used as a reference to calculate the transect width as well as to measure the colony size. Navigation data from a Kongsberg Simrad USBL system type HPR 410P, a Simrad dGPS instrument and a Furuno satellite compass were integrated in the software package Olex to provide ROV navigation and transect position data.

A video frame was extracted every 5 s from the video footage using the software VLC7. This extraction frequency is based on an average speed of 0.7 knots, meaning that a frame is analysed every ~ 1.8 m along the transect. The average surface area covered per image for each dive was calculated using the calibrating tool in Coral Point Count (see below) (Kohler and Gill 2006). This tool calculated the average maximum width and height for each image. The calculation was based on the 5-cm separation between the two laser points. Each frame covered an approximate area of 1.4 m (width) × 0.80 m (height) with a resolution of 1280 × 720 pixels. A total of 322 frames were extracted for analysis (158 frames were extracted from dive 1, 68 from dive 2 and 96 from dive 3; Table 3).

**Table 3:** Overview of the number of frames extracted and used for statistical analyses

Dive	Number of extracted frames	Number of 25-m samples with > 9 frames	Total number of frames used for analyses
1	158	7	148
2	68	3	53
3	96	6	60

### Spatial extent of the different substrate types: *Lophelia pertusa*, *Geodia* sp. and *Mycale lingua*

**Fig. 2:**
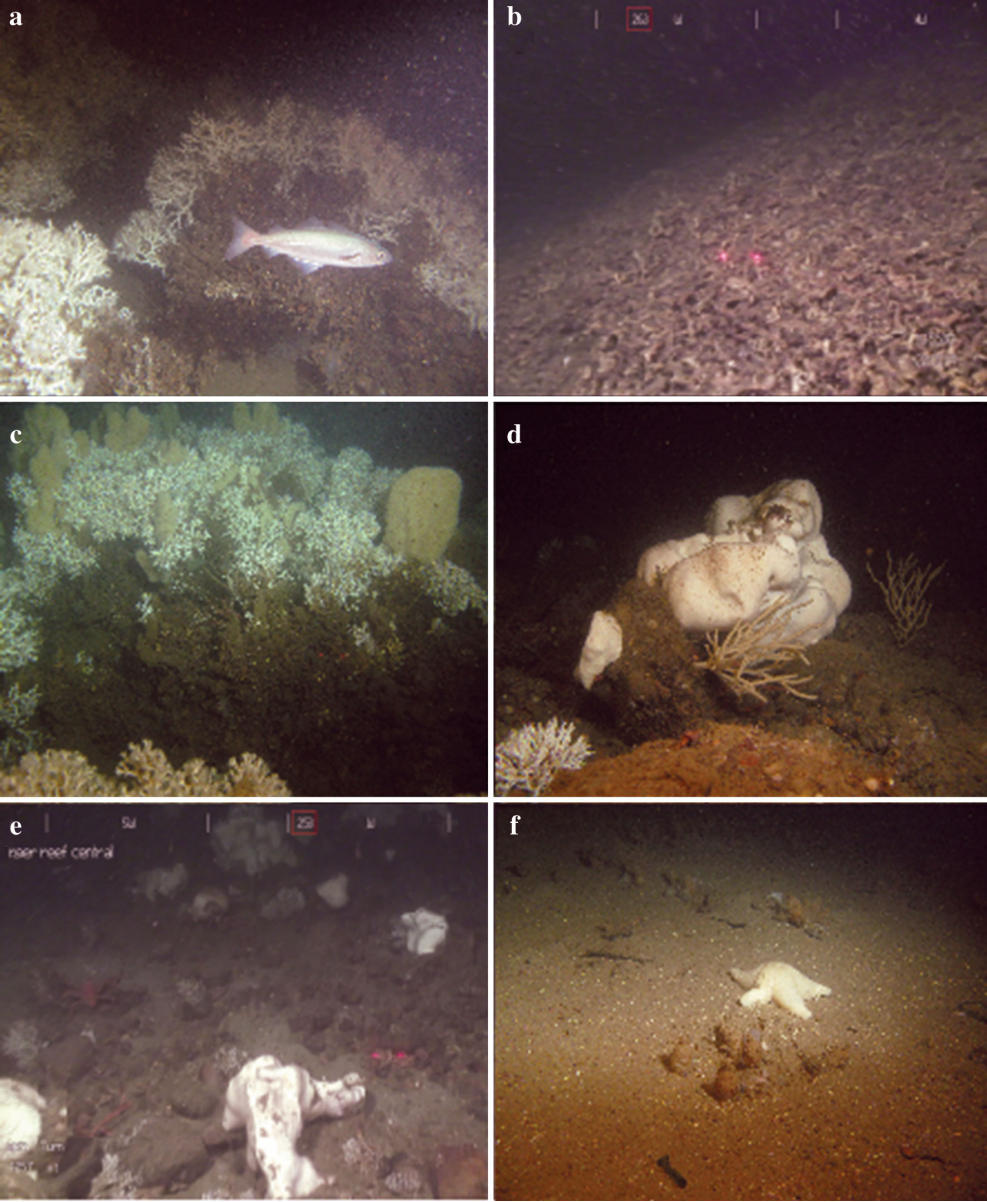
Overview of the habitats documented in the video transects on Tisler Reef. **a** Live *Lophelia pertusa* thickets on top of the dead *L. pertusa* base layer. **b** Coral rubble. **c** *Mycale lingua* growing within *L. pertusa* branches. **d** *Geodia* sp. **e** hard substrate colonized by several *Geodia* sp. specimens. **f** Soft sediment with a starfish

The percentage cover of the substrate types (coral rubble, soft substrate and hard substrate), the coral colonies (live and dead *L. pertusa*) and sponges (*Geodia* sp., *Mycale lingua*) (Fig. 2) was calculated with a 50-point quadrat method using the software Coral Point Count with Excel Extensions 4.1 (CPCe) (Kohler and Gill 2006). This CPCe software is freely available, user-friendly, time efficient and provides reliable results for the percentage cover calculations of seabed organisms and substrates. The points were randomly placed over each image, and the species and substrate below each point were noted. In the CPCe software, the percentage cover can be calculated for an individual image or for a group of images.

### *Lophelia pertusa* morphology

**Fig. 3:**
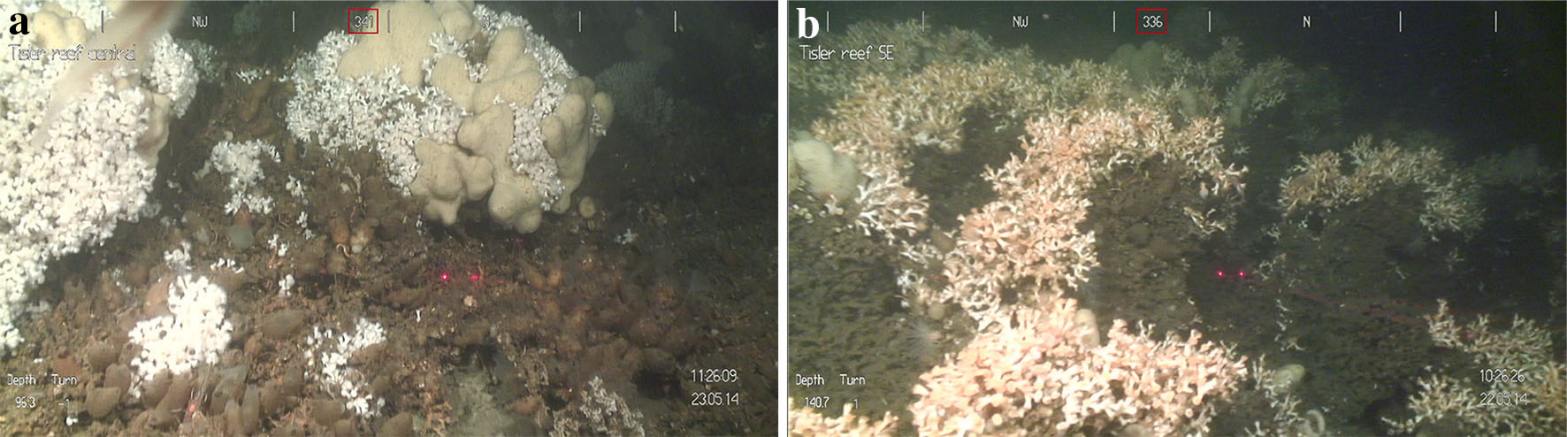
**a** *Lophelia pertusa* large “cauliflower” morphotype. **b** Medium-sized *L. pertusa* “bush-like” coral colonies. Laser scale: 5 cm

**Fig. 4:**
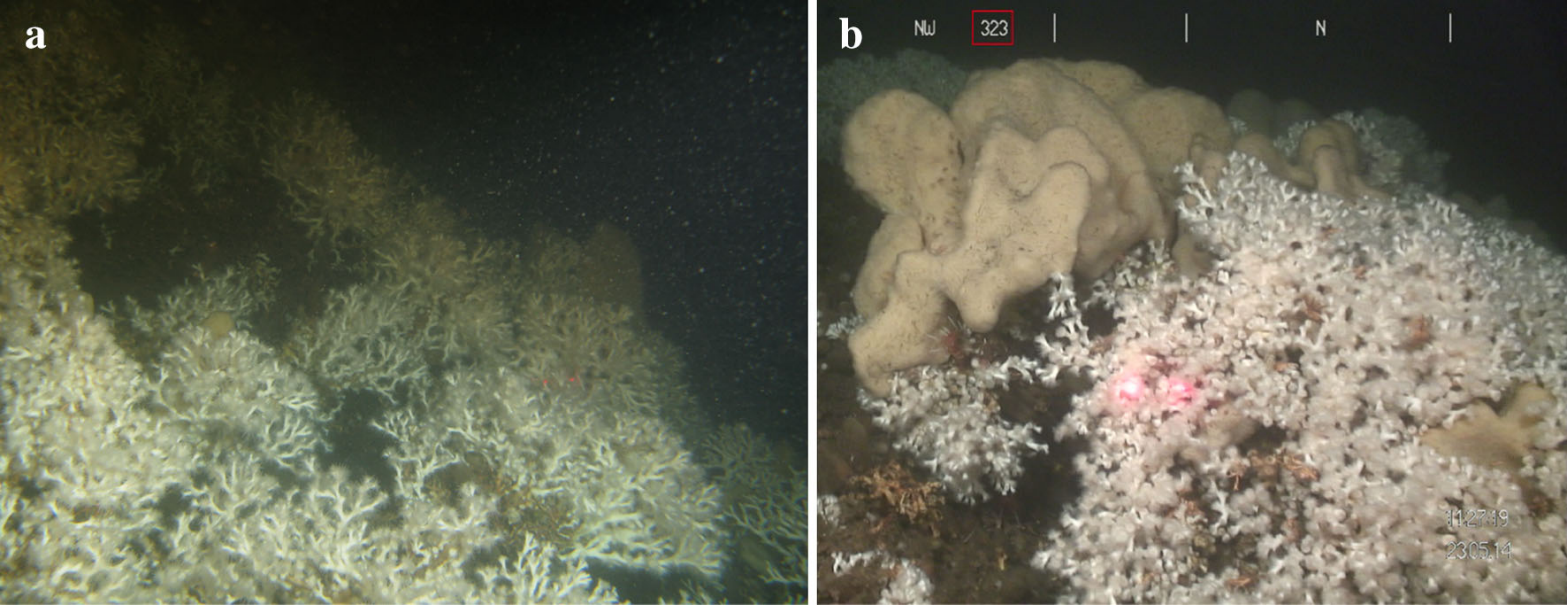
**a** *Lophelia pertusa* with “long” branches. **b** *L. pertusa* with “short” branches. Laser scale: 5 cm

**Fig. 5:**
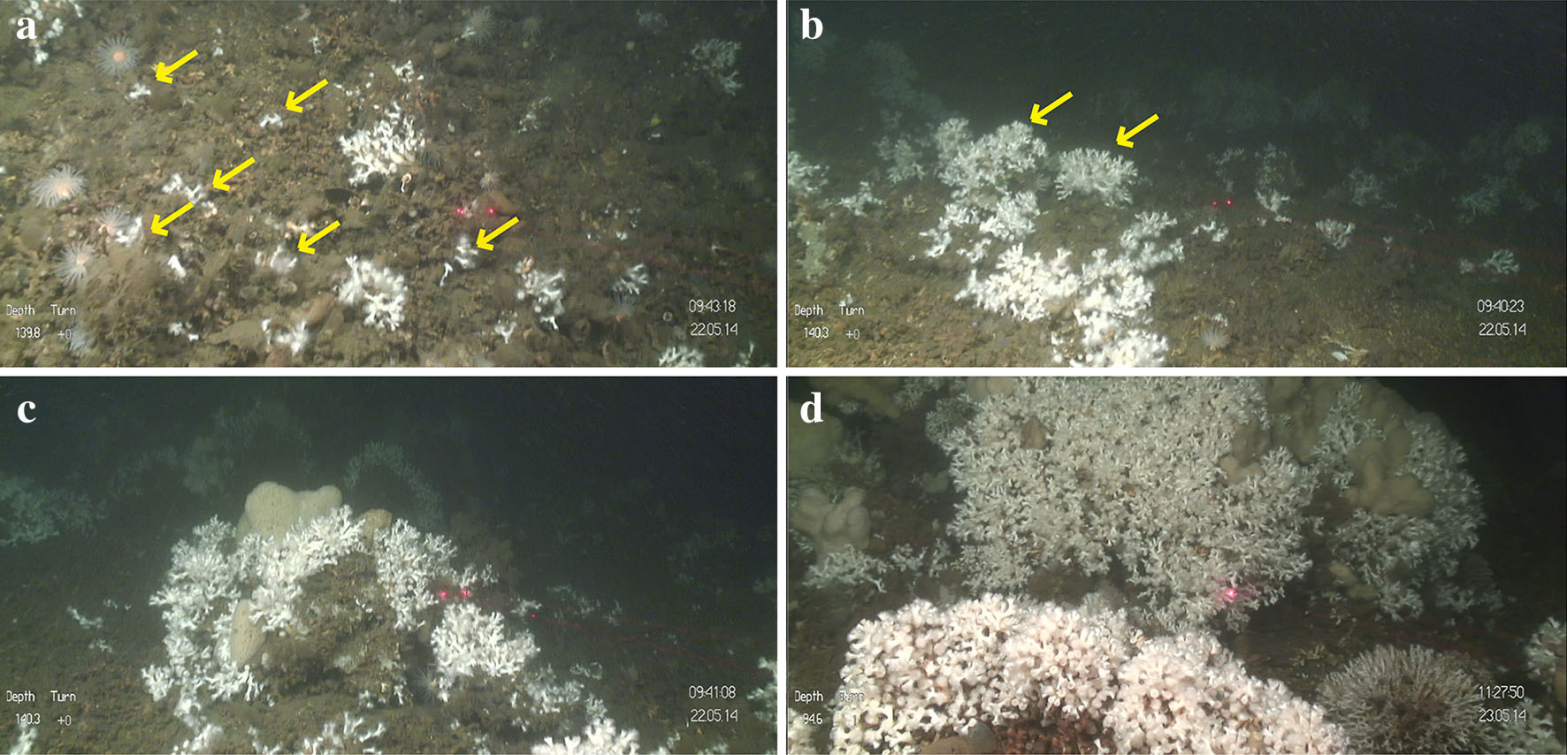
The different size classes defined for *Lophelia pertusa* in this study: **a** very small (< 5 cm); **b** small (5–30 cm); **c** medium (30–100 cm); and **d** large (> 100 cm). Laser scale: 5 cm. The yellow arrows indicate the very small (**a**) and small (**b**) coral colonies

The morphology of coral colonies was described as a function of their overall shape, branch length and colony size. Past studies have identified two dominant morphology classes of *L. pertus*a. The first is a compact “cauliflower” shape, which results from live coral branches growing symmetrically in multiple directions (Freiwald et al. 1999; Rogers 2004). The second morphology class has a “bush-like” shape with colonies that grow in a more unidirectional plane (Wilson 1979; Chindapol et al. 2013). Here, these two morphotypes were used as a first category distinguishing the colony’s shape as (1) cauliflower versus (2) bush-like (Fig. 3). The colony’s overall branch morphology was identified as (1) short (< 5 cm) versus (2) longer branching patterns (> 5–10 cm) (Fig. 4). The category size included very small (< 5 cm), small (5–30 cm), medium (30–100 cm) and large (> 100 cm) coral colonies (Fig. 5). In this study, an individual *L. pertusa* colony refers to a distinctive visual colony. Skeletal fusion in *L. pertusa* is common (Hennige et al. 2014), and therefore, a “colony” as termed here may represent multiple genotypes.

### Statistical analyses

For the statistical analyses, each dive was divided into 25 m sub-transects. This length was chosen as it gave the best representation of the variability in the data. A proportion of the frames were excluded from the analyses due to overlap or low visibility. The number of frames that were of good quality for analyses varied among dives (Table 3), meaning that some of the 25 m sub-transects did not contain enough analysed frames to produce reliable and representable results. Only 25 m sub-transects with a minimum of nine good frames were retained in the analyses.

A 0.5 log_10_ (1 + *x*) transformation was applied to the percentage cover data of the substrate classes *L. pertusa* (live and dead) and the sponges (*Geodia* sp. and *M. lingua*). This transformation decreases the relative importance of high percentage coral cover values (Guinan et al. 2009). This transformed dataset was also used to investigate the co-occurrence of the sponges (*Geodia* sp. and *M. lingua*) and *L. pertusa* (live and dead), for which the Pearson product-moment correlation coefficient (*r*) was calculated in R. Since different morphology classes (shape, branch length, size) were attributed to every coral colony, density was used instead of percentage cover. The densities of *L. pertusa* morphology classes were calculated by dividing the number of colonies by the average surface area per sub-transect.

Statistical analyses used the PRIMER 6 software package (Clarke and Warwick 2001). The aim was to establish whether there was a difference in CWC distribution and morphology between the 25-m samples from the transects according to their location on the reef (SE vs. the NW side). In PRIMER, a Bray–Curtis resemblance matrix was used in analyses of similarity (ANOSIM) to test whether there were significant differences between the samples from the SE and NW side of the reef. SIMPER analyses were then carried out to identify how the percentage cover (*L. pertusa, M. lingua, Geodia* sp. and the different substrates) and *L. pertusa* morphology classes differed between the different locations.

## Results

### Hydrodynamic data: current direction and speed

**Fig. 6:**
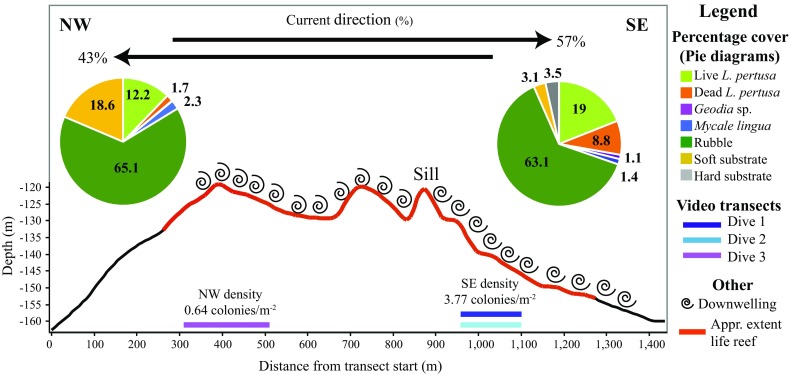
Diagram of Tisler Reef based on Fig. 2 in Wagner et al. (2011); red line shows the approximate extent of the live reef, and swirls show location of downwelling. The values along the *x*-axis represent the distance from the NW end towards the SE end of the reef. The location of the video transects is indicated by the coloured lines on the bottom of the figure. The black arrows in the top of the figure represent the percentage of the current direction. The pie diagrams contain the average percentage cover of live *Lophelia pertusa*, dead *L. pertusa*, *Geodia* sp., *Mycale lingua*, rubble, soft and hard substrate for the NW and SE side. The density of live corals colonies is given for both sides (m^2^) just above the location of the video transects

**Fig. 7:**
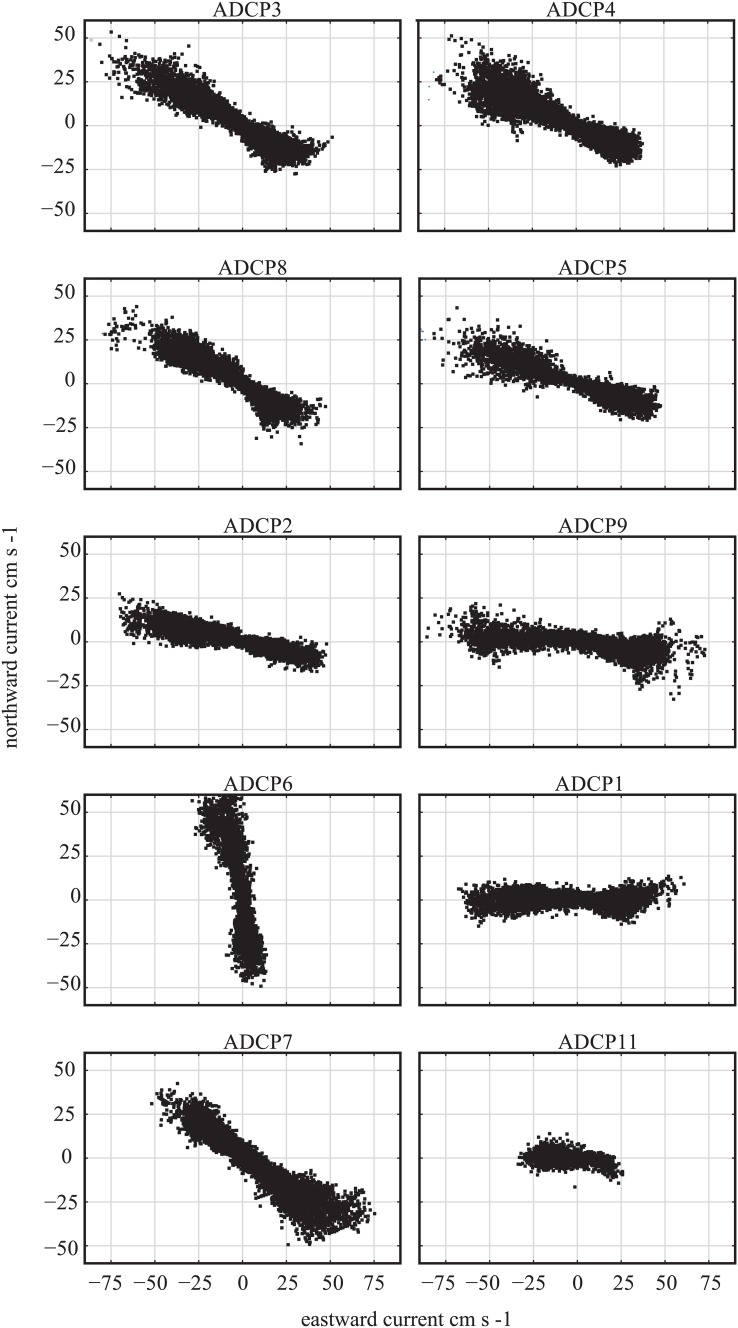
The speed (cm s^−1^) and direction of the main current axis for current meter deployments at 86 m depth

The current flowed towards the SE for 57% of the time and to the NW for 43% of the time in the 4-yr period (Fig. 6). The direction of the flow over the reef typically lasted for several days, sometimes up to two weeks, before reversing. The highest current speed recorded in our dataset was 99.8 cm s^−1^, in the NW direction. Higher current speeds were more frequent when the flow was in the NW direction (Fig. 7). Current speed also varied with the position of the deployments (Fig. 7). For example, ADCP3 (at the NW of the group) had stronger currents towards the NW, while ADCP7 (positioned to the SE) recorded stronger currents flowing SE. This acceleration of near-bed currents downslope, downstream of a sill is characteristic of stratified flow over sills (Farmer and Denton 1985).

### Spatial extent of substrate types

The average percentage of rubble and soft substrates was significantly higher on the NW side, while hard substrates were significantly more prominent on the SE side of Tisler Reef (*R* = 0.384, *p* < 0.01) (Fig. 6). The average percentage of both live and dead *L. pertusa* per 25-m sample was significantly higher on the SE side than the NW side (*R* = 0.57, *p* < 0.01; Fig. 6). This was reflected in the ratio of live:dead *L. pertusa*, which was 2.15 on the SE side and 7.17 on the NW side. This indicates that the percentage cover of live and dead *L. pertusa* on the SE is closer to equal, while on the NW side there is a high percentage cover of live but a low percentage cover of dead *L. pertusa*. The percentage cover of the sponge *M. lingua* was significantly higher on the NW side, while, conversely, the sponge *Geodia* sp. had a higher percentage cover on the SE side (*R* = 0.31, *p* = 0.02; Fig. 6).

Live cover of *L. pertusa* was weakly correlated (*r* = 0.33, *p* = 0.213) with *Geodia* sp. A stronger positive correlation (*r* = 0.67, *p* = 0.004) was found between dead *L. pertusa* and *Geodia* sp. Cover of both live and dead *L. pertusa* was strongly positively correlated with cover of *M. lingua* (*r* = 0.69, *p* = 0.003 and *r* = 0.70, *p* = 0.002, respectively).

### *Lophelia pertusa* morphology

**Fig. 8:**
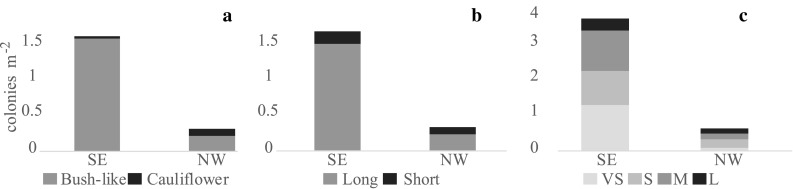
Density (colonies m^−2^) of colonies with different shape categories, branch length and the different size classes on the SE and NW sides of the reef. **a** Cauliflower-shaped versus bush-shaped colonies. **b** Short versus long coral colony branches. **c** Proportion of size classes. *VS* very small, *S* small, *M* medium and *L* large

In total, 1708 coral colonies were counted. The density of corals was significantly lower on the NW side (0.64 corals m^−2^) than on the SE side (3.77 corals m^−2^) (Fig. 6). Very small (*n* = 544) and small (*n* = 463) colonies had on average between 1 and 50 branches which were not large enough to visually assign a morphology class. Therefore, only the medium (*n* = 527) and large (*n* = 174) colonies were described as a function of shape and branch length. There were significant differences in coral morphology between the NW and SE side of the reef (shapes: *R* = 0.63, *p* < 0.01; branch length: *R* = 0.58, *p* < 0.01; size classes: *R* = 0.54, *p* < 0.01) (Fig. 8). All morphology classes were more abundant on the SE side of the reef, but the proportion of the different morphologies differed. The NW side had a higher proportion of cauliflower-shaped colonies than bush-like colonies (SE: 0.02 vs. NW: 0.43). The NW side also had a larger proportion of short branches versus long branches (SE: 0.12 vs. NW: 0.43). The proportion of very small colonies was much higher on the SE side (0.52) than the NW (0.14).

## Discussion

### Percentage cover of *Lophelia pertusa*

On Tisler Reef, CWC growth and development takes place on both sides of the sill, due to downwelling occurring on both sides (Fig. 1c; Wagner et al. 2011). However, there was significantly lower coral cover on the NW side of the reef. Firstly, the higher percentage cover of soft substrates on the NW side is likely to decrease the availability of hard substrates that corals need for settlement (Wilson 1979). Secondly, the dominant current flows to the SE 57% of the time and to the NW 43% of the time, which broadly confirmed the pattern observed over a 2-yr period by Wagner et al. (2011). This implies that the SE side of Tisler Reef is exposed more frequently and for longer periods of time to downwelling, where warmer and more chlorophyll-rich water is transported to the corals from surface waters, creating more favourable growing conditions for *L. pertusa* and sponges, similar to other areas (Duineveld et al. 2007). These two factors could explain why the SE side had a higher percentage of coral habitat.

Tisler Reef is a highly dynamic environment. Current speeds as high as 99.8 cm s^−1^ were recorded during 2006–2010, higher than previously reported (Wagner et al. 2011). Suspension- and filter-feeding organisms, like corals and sponges, thrive in high-current-speed environments, as it increases their food encounter rates (Best 1988; Hunter 1989; Fabricius et al. 1995; Sebens et al. 1998). Fast currents also help prevent polyps from becoming clogged with sediments (Brooke et al. 2009; Larsson and Purser 2011). Zooplankton capture rates can vary among coral species and can depend on flow speed, water temperature and prey size (Purser et al. 2010; Gori et al. 2015; Orejas et al. 2016). For *L. pertusa*, the zooplankton capture rate is optimal at slower speeds of 2.5 cm s^−1^ (Purser et al. 2010). When the current exceeds this speed, the polyps could bend backwards which reduces the feeding surface (e.g. gorgonians: Fabricius et al. 1995; tropical scleractinians: Sebens and Johnson 1991). At higher current speeds, prey could also escape from the polyps as the higher flow velocities may give them sufficient momentum to break free (Purser et al. 2010). Optimal conditions for coral feeding, and therefore growth, can change constantly within a reef as the local current speed and direction change not only at large timescales but also on a daily basis. For instance, at Mingulay Reef, where *L. pertusa* is also the dominant reef-forming coral, current speed varies from ~ 2 to ~ 30 cm s^−1^ in a single day (Davies et al. 2009). On coral reefs, dense coral thickets slow down current speeds due to friction with their framework (Roberts et al. 2009). Therefore, in highly dynamic environments, like Tisler Reef, the optimal conditions for coral feeding will vary over time for different individual coral colonies, i.e. many corals will experience “optimum” conditions at some point during a day. It is likely that, depending on the current direction, the SE and NW sides are exposed to different current speeds that could affect CWCs’ ability to feed and grow. Unfortunately, our data could not be used to compare current speeds between the NW and SE sides of the reef since differences in the measured current speed were observed even though the ADCP deployments were relatively close to each other. The measured current speeds tended to be stronger in sites further towards the NW (Fig. 6). This is because, close to the seabed, flow over a sill will tend to decelerate upstream and accelerate downstream.

### *Lophelia pertusa* morphology

Corals on a reef are frequently subjected to different flow directions and changes in environmental variables that affect their growth morphology (Wainwright and Dillon 1969; Mortensen and Buhl-Mortensen 2005; Todd 2008; Chindapol et al. 2013). When corals occur in an area with strong unidirectional currents, colonies develop a more bush-like shape with their branches growing in the upstream direction (Chindapol et al. 2013). At Tisler Reef, the water currents are forced through the sound between the Tisler and Hvaler Islands. Consequently, the currents are relatively unidirectional resulting in the observed higher density of asymmetrical, bush-like coral colonies. Cauliflower-shaped colonies were less abundant on Tisler Reef. This symmetrical shape is more likely to develop under low current speeds (Chindapol et al. 2013). Areas where this shape was observed could therefore indicate more sheltered conditions within the reef.

The proportion and abundance of very small colonies were significantly higher on the SE side of Tisler Reef, which indicates recruitment. This could be a consequence of the more optimal feeding conditions due to more frequent periods of downwelling, allowing coral larvae to survive and grow. Alternatively, the higher percentage cover of live and dead coral structures may increase the settlement of larvae. The structure of the live and dead corals increases turbulence (Chamberlain and Grauss 1975; Hennige 2016), which could allow *L. pertusa* larvae to enter the bottom boundary layer to access rubble and other hard substrates for attachment. The planula larvae of *L. pertusa* seems to prefer settling on protruding bodies such as in between coral rubble or even on oil rigs (Wilson 1979; Bell and Smith 1999; Gass and Roberts 2006). Live *L. pertusa* is not a suitable settling substrate as the permanent mucus layer (coenosarc) on their skeleton prevents attachment of sessile epibiotic species (Freiwald 2002; Buhl-Mortensen et al. 2010). However, diverse microhabitats are provided by dead coral skeletons which facilitate the high biodiversity associated with reef-forming CWCs (Mortensen and Fosså 2006; Buhl-Mortensen et al. 2010). The NW side of the reef has coral rubble present, but lacks large amounts of dead *L. pertusa* framework. Together with the higher percentage cover of soft substrates on the NW side, insufficient settling substrates, less turbulence and elevation out of the bottom boundary layer could explain why fewer small colonies were observed on the NW side of Tisler Reef (Masson et al. 2003; Strömberg 2016) (Fig. 2b). Thus, the presence of the structure formed by coral colonies, in this case on the SE side of Tisler Reef, may have a positive effect on the suitability of the area for the larvae.

### Percentage cover of sponges

*Geodia* sp. had lower percentage cover than *M. lingua*. The low presence of this sponge at Tisler Reef could be a consequence of interspecific competition between *Geodia* sp. and *L. pertusa* for settling substrate and food. This was also indicated by the positive correlation between the cover of dead *L. pertusa* and *Geodia* sp. Purser et al. (2013) posited that competition for substrate may be one of the reasons for the low co-occurrence of *L. pertusa* and *Geodia baretti.* The low percentage cover of *Geodia* sp. on Tisler Reef could also be caused by periods where the maximum temperature was 1.5–3 °C higher than the normal maxima (~ 9 °C). These high temperatures recorded at shallow depths between 90 and 120 m have resulted in mass mortality events (HERMES 2008; Guihen et al. 2012). The depths at which the high temperatures were recorded fall within the depth range at which the transect on the NW side was collected. Interspecific competition, exposure to high temperatures and the slow growth rate of *Geodia* sp. could therefore explain the low presence of this sponge at Tisler Reef.

A positive correlation between the cover of live and dead *L. pertusa* and the occurrence of *M. lingua* was found at Tisler Reef. This relationship varies at different reefs; a positive correlation was documented at Røst Reef (Norway), whereas a negative correlation occured at Sotbakken and Traena reefs (Purser et al. 2013). In contrast to *Geodia* sp, *M. lingua* can use living *L. pertusa* as a substrate; they can grow within a colony (Lavaleye et al. 2009; Purser et al. 2013) and are therefore not in direct competition for hard substrate for settlement (Figs. 2c, 3a, 4b, 5c, d). This could explain why the percentage cover of *M. lingua* is much higher than that of *Geodia* sp. Interestingly, a higher percentage cover of *M. lingua* was found on the NW side of Tisler Reef. This finding seems surprising, as a higher percentage of *L. pertusa*, on which *M. lingua* can grow, was recorded on the SE side (Figs. 2c, 3a, 4b, 5c, d). It is possible that the corals occurring on the NW side were exposed to higher rates of stress, giving *M. lingua* a competitive advantage (Rützler and Muzik 1993). Tisler Reef is a shallow, nearshore reef; it is likely to be exposed to seasonal temperature fluctuations, terrestrial and human-induced influences and more eutrophic conditions caused by river outflow and agricultural activity (van Soest and De Voogd 2015). Studies on intraspecific interactions and environmental stressors could provide insight into what drives this difference.

### Importance of this study

This study highlights differences in the spatial distribution of live versus dead coral framework and morphology within a reef, which are mostly related to variations in the substrate and local hydrodynamics. Buhl-Mortensen et al. (2010) and Wheeler et al. (2007) provided illustrations and acoustic data that gave an indication of the distribution and abundance of live versus dead *L. pertusa* in a CWC reef or on a CWC carbonate mound. De Clippele et al. (2016) showed the presence of live and dead coral framework on small reefs in Mingulay Reef by using microbathymetry (35 × 35 cm cell size). These studies showed that live coral grow into the dominant current to optimize food capture. On Tisler Reef, the dominant current direction reverses, and therefore, Wagner et al. (2011) observed live coral growth on both ends of the reef. Even though live coral was present at both ends, clear differences in the percentage cover of live and dead coral were observed in this study. Wheeler et al. (2007) highlight that environmental controls such as current dynamics, temperature, salinity, pH, food supply and sediment supply affect the growth and thus the morphology of CWC carbonate mounds. Wheeler et al. (2007) indicated that the morphology of mounds can provide clues to the environment. The difference between CWC mounds and reefs is the shallow nature and relatively young age of reefs, which creates a more dynamic and unpredictable environment compared to CWC mounds. Our study indicates that the local hydrodynamics and food supply affect the reef’s growth. Studies describing the morphology and fine-scale distribution of the different habitats provided by corals, help to understand how CWC reefs grow. Dead coral framework and coral rubble provide a large variety of microhabitats that can be used by, for example, crustaceans, crinoids, other corals, fish and microorganisms (Costello et al. 2005; Buhl-Mortensen et al. 2010; Henry and Roberts 2017). This study showed that understanding the variation in the amount of live versus dead coral framework is complex but likely related to differences in fine-scale hydrodynamic processes and food supply. Mapping differences in live versus dead coral framework and rubble can shed light on coral recruitment success within a reef and the distribution of associated organisms.
